# The use of quantitative electroencephalography in evaluating epilepsy treatment

**DOI:** 10.1186/s42494-026-00267-1

**Published:** 2026-07-07

**Authors:** Jingjing Qiu, Yixia Chen, Zhiyuan Ning, Bing Zhang, Bo Shen, Yuying Zhao, Ying Liu

**Affiliations:** 1 Huzhou Central Hospital, The Fifth School of Clinical Medicine of Zhejiang Chinese Medical University, Huzhou, Zhejiang 313000 P. R. China; 2 The Fifth School of Clinical Medicine, Zhejiang Chinese Medical University, Hangzhou, Zhejiang 310053 P. R. China

**Keywords:** Quantitative electroencephalography, Therapeutic assessment, Anti-seizure medications, Epileptic source localization, Functional connectivity

## Abstract

Because quantitative electroencephalography (QEEG) presents objective, data-driven measurements of brain activity with better performance than conventional EEG, it has become more popular for evaluating therapeutic response in epilepsy. Data from over 160 English-language papers on the use of QEEG in the evaluation of epilepsy treatment that were published up to March 2026 were assessed in this review. This review includes anti-seizure medications (ASMs), long-term EEG monitoring, neuromodulation methods such as vagus nerve stimulation (VNS), epilepsy surgery and epileptogenic zone identification, and new models using AI. Instead of providing a methodical or completely reproducible synthesis, this review concentrates on critically evaluating methodological trends, translational significance, and current limitations in various applications. The significant differences in patients’ demographics, EEG acquisition methods, analytical approaches, and clinical goals caused difficulties in a thorough systematic review or meta-analysis. However, a qualitative review of the literature showed recurrent QEEG patterns associated with treatment response, areas of conflicting evidence, and the translational readiness of different metrics. Studies frequently find correlations between changes in spectral power, functional connectivity (FC), and epileptiform activity indices (EAI) and clinical outcomes. However, the reproducibility and robustness of these correlations greatly vary depending on treatment modality and methodological environment. Hence, the potential of QEEG in directing customized treatments is limited by the lack of standardized procedures, normative databases, and prospective validation. All things considered, it appears most promise for long-term therapy tracking and, in certain situations, for enhancing conventional clinical evaluation. This review emphasizes the necessity of thorough studies that prioritize practical application, clinical interpretability, and repeatability. Additionally, it highlights the current limitations and therapeutic potential of QEEG-based treatment evaluation.

## Background

Electroencephalography (EEG), which captures the electrical activity of the brain, is mainly used to diagnose neurological disorders [1]. EEG is primarily utilized in clinical practice to identify abnormal waveforms, such as interictal epileptiform discharges and changes during epileptic seizures, by visual evaluation with the assistance of trained professionals [2, 3]. The study conducted by Berger et al. mostly concentrated on changes in EEG signals in the temporal domain [4].

With the increasing use of computers to analyze digital EEG signals, the field of quantitative EEG (QEEG) has expanded [5]. In this field, computational and mathematical methods are used to process, convert, and interpret digital EEG data. Each sampling point can be represented numerically since EEG signals are digital. This makes it possible to use specialized software for systematic signal processing, including automatic event detection, frequency-domain transformation, and artifact removal [6]. As a result, a number of complementary QEEG analytical methods have been developed. Signal complexity (SC) measures nonlinear temporal properties and neuronal synchronization [7], power spectral analysis characterizes oscillatory activity distribution in frequency bands and reflects large-scale cortical dynamics [7], functional connectivity (FC) analysis describes statistical dependencies among spatially distributed EEG signals [8], and network topology metrics (NTM) indicate the global and regional organization of brain networks [9, 10]. By analyzing unique but linked neurophysiological systems, these techniques may yield convergent or divergent results, depending on the analytical framework and clinical conditions.

These days, QEEG is increasingly used to evaluate the effectiveness of treatments for neurological and mental disorders, including epilepsy. Seizure detection, seizure prediction, and epilepsy identification are only a few research domains for QEEG studies in epilepsy [11–13]. Each approach uses various neurological systems with the assistance of spectral power, SC, FC, or NTM. Studies targeting these goals may yield varied and sometimes contradictory results because of differences in EEG signal sources, analytic methods, and outcome criteria [11, 14]. Hence, using QEEG to assess treatment is a unique clinical goal that concentrates on long-term changes in brain activity linked to therapeutic interventions instead of event detection or predictions.

QEEG has shown potential in pharmaceutical response assessment, therapeutic monitoring, and outcome prediction after nonpharmacological therapies. It also enables automated characterization of abnormal EEG patterns [15]. The generalizability and clinical translation of current research are limited by methodological heterogeneity, inconsistent outcome measures, and inadequate validation. The current review concentrates on the function of QEEG as a longitudinal tool for assessing treatment response in pharmacological, surgical, neuromodulatory, and emerging computational interventions, in contrast to previous narrative or systematic reviews that mainly concentrate on the diagnostic potential of QEEG in epilepsy. In this regard, the current review summarizes the available data, clarifies methodological variations, and differentiates exploratory findings from those that approach clinical usefulness in the assessment of epilepsy treatments.

The quantitative assessment of treatment-related changes in EEG-derived metrics, including seizure burden, electrophysiological background modulation, network reorganization, and functional improvement that are interpreted in connection to clinical outcomes, is referred to in this review as treatment assessment.

## Methods

### Review design

In order to provide a clinically focused synthesis of the application of QEEG in assessing epilepsy treatment, this study was carried out as a narrative review. The review was performed using a well-defined study selection procedure and an organized literature search to increase transparency and reproducibility. Mapping important analytical domains, methodological trends, and translational considerations in several study designs and clinical situations are the main goals of the review.

The goal was to identify representative and thematically relevant studies that added to our current understanding of how QEEG-derived features have been used to assess treatment-related outcomes in epilepsy, with particular attention to spectral, epileptiform, complexity, connectivity, and network-based measures. The primary QEEG analytical domains and representative features discussed in this review are presented in Table 1. These metrics provide a conceptual framework for understanding QEEG results in the evaluation of epilepsy treatment.

Because this review is narrative, the selection of research was based on methodological representativeness, clinical utility, and topic significance. Nonetheless, an effort was made to improve transparency by providing a detailed description of the literature search strategy and research selection process, and key elements are presented in a PRISMA-style flow diagram.

**Table 1:** Representative QEEG features and analytical domains discussed in this review

QEEG Domain	Conceptual Description	Representative Metrics
Power spectrum analysis (PSA) [16]	Quantifies the distribution of EEG signal power across frequency bands, reflecting large-scale cortical activation patterns and treatment-related spectral shifts.	Absolute and relative band power; power ratios
Epileptiform activity indices (EAI) [17]	Quantitative measures of interictal epileptiform discharges, capturing the burden and temporal density of pathological EEG activity relevant to treatment response.	Spike frequency; proportion of recording with epileptiform discharges
SC analysis [7]	Assesses the nonlinear and dynamic properties of EEG signals, providing insight into changes in cortical signal variability and organization associated with disease state and treatment effects.	Sample entropy; fractal dimension; Hurst exponent
FC analysis [18]	Evaluates statistical dependencies between EEG signals from different brain regions, reflecting alterations in functional integration and network synchronization related to epileptic activity and therapeutic modulation.	Coherence; phase synchronization indices; Granger causality–based measures
NTM [19]	Applies graph-theoretical frameworks to characterize large-scale EEG network organization, supporting assessment of network reconfiguration and localization of epileptogenic regions in treatment contexts.	Small-worldness; node degree; centrality measures

### Literature search strategy

To find English-language research published up to March 2026, a systematic literature search was carried out across PubMed, Web of Science Core Collection, IEEE Xplore, and ScienceDirect. The combined search terms for QEEG, treatment evaluation, and epilepsy are presented as follows:

(epilepsy OR “epileptic seizures”) AND (treatment OR therapy OR intervention OR “pharmacological response” OR “surgical outcome”) AND (“quantitative EEG” OR QEEG OR “EEG spectral analysis” OR “EEG connectivity”).

**Fig. 1:**
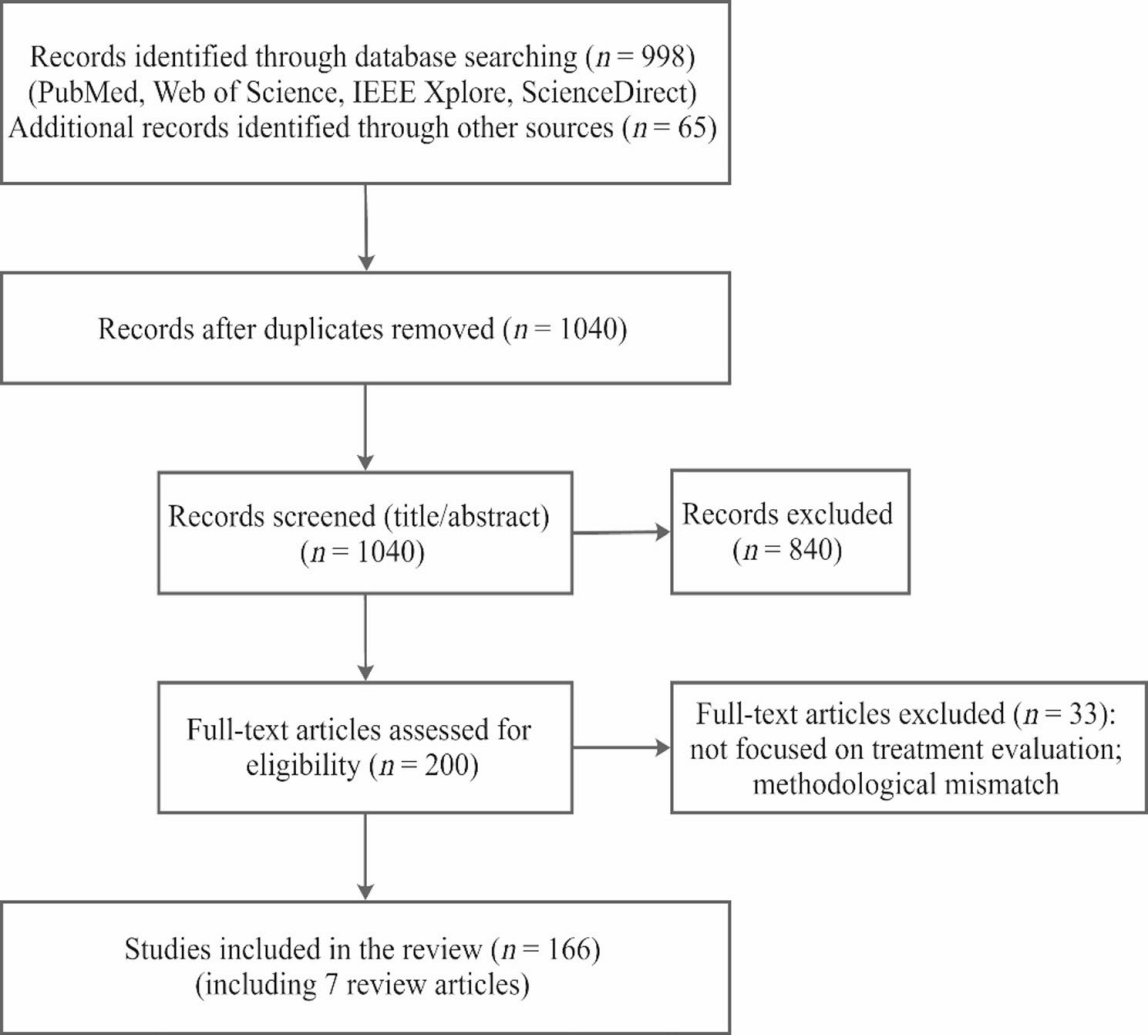
PRISMA-style flow diagram of literature identification, screening, eligibility assessment, and study inclusion. Figure 1 illustrates the general process of literature identification and selection adopted for this critical narrative review with a scoping approach. The process was intended to provide an overview of the existing evidence rather than to achieve exhaustive study inclusion

We performed an additional search using the following terms: (epilepsy) AND (“quantitative EEG” OR QEEG) AND (“machine learning” OR “artificial intelligence” OR “deep learning”) in order to find studies that used AI-based analysis techniques. Figure 1 shows the general procedure for identifying eligible studies.

### Study selection and eligibility

Studies that evaluated treatment-related outcomes, such as responsiveness to anti-seizure medications, neuromodulation, or epilepsy surgery, and used QEEG-derived metrics, including spectral, epileptiform, connectivity, network-based, or nonlinear features, were considered eligible. Studies that had no obvious connection to therapy evaluation and only concentrated on seizure detection or prediction were excluded. Original research articles and relevant reviews supplemented the methodological and conceptual discussion.

Formal risk-of-bias assessment methods were not employed since study designs, patient demographics, and analytical frameworks varied. Alternatively, methodological flaws and other sources of uncertainty were discussed in the limitations and summary sections.

### Data extraction and synthesis

Data on the type of epilepsy, patient demographics, QEEG characteristics, analysis techniques, and treatment-related outcomes were extracted each included study. The results were combined qualitatively due to the variety of study designs, EEG acquisition procedures, and outcome measures.

The synthesis highlights translational significance, methodological robustness, and physiological interpretability while paying attention to areas of overlap, divergence, and uncertainty in the research.

## Anti-seizure pharmacotherapy

QEEG can be used to observe the effects of drugs on brain activity to evaluate the efficacy and side effects of anti-seizure medications (ASMs) and to characterize their impact on the brain’s functional state from a new perspective [20].

### Power spectrum analysis

One of the most well-known QEEG techniques is power spectrum analysis (PSA), which provides a frequency-domain description of brain activity and is still often used in epilepsy studies. To measure oscillatory power throughout the cortex, PSA breaks down EEG data into conventional frequency bands, such as delta (δ), theta (θ), alpha (α), beta (β), and gamma (γ) ranges [16]. PSA offers a repeatable paradigm for evaluating large-scale cortical dynamics and their possible alteration in response to therapeutic interventions by monitoring the distribution of EEG power.

PSA is frequently used in a number of methodological variations, each with unique advantages and disadvantages (Table 2). Although the Fourier Transform (FFT) allows for fast computing and is ideal for long-duration, full-band spectral analysis, its sensitivity to transient or non-stationary events is limited by the assumption of signal stationarity.

The detection of brief epileptiform discharges and high-frequency oscillations (HFOs) is made easier by time-frequency approaches like wavelet analysis, which offer simultaneous temporal and spectral localization. However, this comes at the expense of higher computational requirements and sensitivity to parameter selection [21]. Although careful filter design is necessary to reduce phase distortion and edge effects, filter-bank approaches are simple to implement and provide targeted study of specific frequency ranges. These methodological variations may help to explain some of the heterogeneity found in PSA-based therapy studies and lead to diversity in practical applications.

**Table 2:** Characteristics, advantages, and limitations of power spectral analysis methods and their common applications

Power Spectral Analysis Method	Advantages	Limitations	Common Applications
FFT [22]	Rapid computation and easy implementation; suitable for comprehensive analysis of long-duration EEG recordings.	Assumes signal stationarity; inadequate for detecting transient or non-stationary events.	Widely used for estimating spectral power in long-duration EEG recordings, particularly in conventional frequency band analysis.
Time–Frequency Analysis (e.g., Wavelet) [23]	Provides both temporal and frequency localization; capable of capturing brief discharges and HFOs.	Results may be sensitive to the choice of mother wavelet and scale parameters, requiring careful optimization.	Widely applied for time–frequency analysis of non-stationary EEG signals, including transient discharges and HFOs.
Filter-Bank Methods [24] (Band-pass/High-pass/Low-pass)	Flexible targeting of specific frequency bands; straightforward to implement.	Prone to phase distortion and edge effects; requires careful filter design.	Used to decompose EEG signals into predefined frequency bands, commonly applied in both conventional analyses and machine learning (ML) pipelines.

Instead of isolated changes within a particular frequency band, PSA results across treatment studies typically reflect shifts in the balance between slow- and fast-frequency activity. Changes in relative band power, power ratios, or interhemispheric asymmetry are frequently used to characterize treatment-related EEG modulation, which together represent changes in the global cortical state linked to seizure control and drug effects [25]. EEG abnormalities may also be seen in non-responders, but these abnormalities may not always correspond with clinical improvement or a decrease in seizures. Despite the widespread use of traditional delta-gamma band classifications, their interpretation in treatment evaluation is still primarily phenomenological. Changes in network synchronization or altered excitation-inhibition balance are examples of underlying neurophysiological mechanisms that are inferential rather than explicitly observable [26].

Different spectral modulation patterns, such as relative slowing or acceleration of background rhythms, have been linked to various anti-seizure medications. However, depending on the type of epilepsy, duration of therapy, and analytical technique, reported effects differ significantly between studies [27–34]. Therefore, rather than being drug-specific electrophysiological signatures, PSA-derived spectral shifts are better understood as markers of treatment-related cortical modulation.

High-frequency EEG activity and HFOs have recently been analyzed using PSA as possible indicators of epileptogenicity and treatment response [35]. The widespread use of high-frequency spectral measurements in therapy evaluation is still limited by technical restrictions, signal-to-noise considerations, and methodological variation among studies, despite their conceptual appeal [36, 37].

Importantly, treatment-related spectral changes are dynamic and may vary over time. Therefore, when interpreting QEEG results linked to ASMs, it is important to distinguish between acute EEG effects observed after single-dose or short-term administration and chronic changes that emerge with continuous treatment. Acute effects often reflect temporary pharmacological regulation of neuronal excitability and oscillatory activity, whereas chronic therapy is more often associated with long-term seizure control and sustained network reconfiguration [38, 39]. Longitudinal investigations, which are influenced by factors such as genetic background, network organization, and the cause of epilepsy, further emphasize significant interindividual heterogeneity [40–43]. A few investigations have linked spectral modulation to treatment-related cognitive effects beyond seizure control, with particular attention to changes within the alpha band [44–46] (Table 3), highlighting the broader therapeutic importance of PSA findings in epilepsy treatments. These reported patterns are representative rather than drug-specific because patient populations, treatment modalities, and analytical methods differ between studies.

**Table 3:** Representative patterns of treatment-related spectral modulation reported in PSA studies in epilepsy

Spectral modulation pattern	Description	Typical analytical expressions
Relative spectral slowing [27–31]	Treatment-associated shift toward increased low-frequency activity, commonly reflected by enhanced delta or theta power accompanied by reduced higher-frequency components. This pattern is frequently reported during early or short-term treatment phases and in contexts associated with increased EEG slowing or sedation-related effects.	Increased relative delta/theta power; decreased alpha/beta power; elevated slow-to-fast power ratios
Relative spectral acceleration [32–34, 47, 48]	Treatment-related enhancement of faster background rhythms, typically characterized by increased alpha and/or beta power with reduced slow-frequency dominance. This pattern has been reported more frequently in patients with favorable seizure control, although variability across studies remains.	Increased alpha and/or beta power; reduced slow-frequency dominance; decreased slow-to-fast power ratios
Redistribution of band-specific power [25, 27, 48]	Selective modulation of individual frequency bands without a uniform shift toward global slowing or acceleration, reflecting heterogeneous cortical responses to treatment across epilepsy subtypes and therapeutic contexts.	Band-specific changes in relative or absolute power without consistent global direction
Altered interhemispheric asymmetry [25, 29, 32]	Treatment-associated changes in lateralized spectral power distribution between hemispheres, potentially reflecting modulation of focal or network-level epileptic activity.	Changes in lateralized power ratios or asymmetry indices

### Epileptiform activity index

Numerous studies have examined variations in the epileptiform activity index (EAI), a quantitative measure of interictal epileptiform load, as a marker for assessing ASM efficacy. However, there is significant methodological heterogeneity in how EAI is calculated [17]. The definition of epileptiform “events” varies among studies, ranging from spike frequency-based thresholds (e.g., ≥ 10 spikes/min) to time-based metrics (e.g., proportion of recording occupied by discharges ≥ 30%). This limits comparability and complicates the development of consistent clinical standards.

Treatment-related suppression of epileptiform activity may be recorded by EAI, as ASM therapy is frequently associated with a decrease in EAI. This trend has been found in both adult and pediatric populations treated with common ASMs, including carbamazepine and valproate [30, 31, 49, 50]. However, these outcomes are inconsistent. In certain epilepsy disorders, particularly benign epilepsy with centrotemporal spikes, there have been reports of paradoxical increases in EAI or persistence of epileptiform activity during sleep after specific therapies [51–54]. Clinical worsening or the advent of novel seizure types has occasionally coincided with this. This heterogeneity emphasizes how EAI changes are context-dependent, indicating differences in drug-specific mechanisms, state-dependent EEG dynamics, and epilepsy subtypes.

The process of standardization has been initiated by international organizations. In order to increase detection reproducibility, the International Federation of Clinical Neurophysiology (IFCN) has presented categorization criteria for interictal epileptiform discharges that incorporate sensor-space and source-space techniques [55]. Technical guidelines have been presented for long-term EEG monitoring by the International League Against Epilepsy (ILAE) and IFCN [56]. Although these programs provide a valuable framework, consistency in EAI measurement is still limited by differences in implementation between institutions.

Dynamic changes in EAI have been investigated as possible markers of seizure risk in addition to therapy response assessment. Variations in the density of epileptiform discharges, such as short-term variability and postictal increases in EAI, have been linked to the occurrence of subsequent seizures [57, 58]. These results imply that rather than acting as a reliable biomarker, EAI might be a reflection of underlying network instability. Nevertheless, there is still little data available, and bigger, prospective studies are needed to confirm the prognostic efficacy of EAI.

### Functional connectivity

Conventional QEEG metrics, such as spectral power, mostly show localized electrical activity, but they offer limited information on the large-scale network interactions that underlie treatment-related brain rearrangement. By describing statistical dependencies between spatially distributed EEG signals, FC analysis expands on QEEG and provides a systems-level view of brain dynamics [59, 60]. FC-based methods have been used to study treatment-related regulation of large-scale neuronal interactions since epilepsy is increasingly understood as a network disorder involving scattered and dynamically interacting regions [61–63].

Methodologically, FC measures cover a wide range of analytical techniques with varying linearity, directionality, and frequency dependency [18]. These can be broadly divided into two categories: directed measurements that try to infer causal or directional interactions between regions, and undirected measures of statistical association (such as coherence and phase synchronization), which quantify the intensity of coupling between signals. Granger causality-based techniques, for instance, provide an estimate of directional functional relationships by determining if prior activity in one area of the brain enhances the prediction of future activity in another. As a result, methods, including frequency band selection, signal preprocessing, and the specific metrics greatly limit the comparability of FC results among studies.

Among studies, treatment-related variations in FC show variability and no discernible directional pattern. Instead of indicating steady increases or decreases in connectivity, these variations appear to rely on the type of epilepsy, the brain’s condition, and the analytical technique. Importantly, both epileptic and non-epileptic conditions have shown enhanced synchronization, particularly in low-frequency bands, indicating that FC variations may reflect global network states instead of disease-specific mechanisms [64, 65]. Increased connectivity is not necessarily harmful. The interpretation of FC variations as direct indicators of therapy success is complicated by this uncertainty.

Clinically, FC-derived metrics can provide supplementary information about treatment-related network-level reorganization. However, significant heterogeneity and inconsistent findings among trials limit their value as stand-alone indicators for treatment response or outcome prediction [33]. Multimodal studies, including combined EEG–functional magnetic resonance imaging (fMRI) studies, which demonstrate that network variations differ among epileptic syndromes and treatment conditions, further highlight this heterogeneity [66, 67].

Hence, FC analysis presents a helpful framework for understanding the network-level effects of epilepsy treatment. However, its current role is still interpretative instead of predictive. Further developments will require methodological standardization, improved reproducibility, and integration with different modalities in order to enhance its therapeutic utility.

## Epileptic source localization

Although FC analyses capture treatment-related interactions at the network level, their spatial interpretability is inherently constrained by sensor-level representations [68]. Source-level approaches, including epileptic source localization, may provide a complementary spatial framework that can facilitate the interpretation of network alterations and electrophysiological reorganization, particularly in the context of surgical or invasive interventions. Methods for localizing epileptogenic zones have evolved alongside advances in QEEG source localization techniques, which have increasingly been explored in treatment evaluation–oriented research contexts. Although terms such as the epileptogenic zone, seizure onset zone, and epileptic focus are sometimes used interchangeably in the literature, they represent overlapping but distinct concepts. In this review, these terms are discussed in a generalized manner for clarity, particularly when referring to source localization and network-level analyses used to characterize treatment-related electrophysiological patterns rather than guiding surgical decision-making.

Early work primarily relied on scalp EEG–based dipole modeling to characterize the sources of sharp waves and seizure activity [69–71]. In order to enhance the anatomical interpretability and spatial characterization of epileptiform activity, further methodological advancements linked three-dimensional source localization frameworks with magnetoencephalography (MEG) and magnetic resonance imaging (MRI) [72–74]. In certain patient cohorts, power imaging of interictal epileptiform discharges has been shown to identify potential epileptogenic regions [75, 76]. Such source-level representations have been employed in treatment evaluation-oriented investigations to investigate whether spatially limited epileptiform generators exhibit persistence or reconfiguration after therapeutic interventions.

Semi-automated and automated procedures have been suggested due to the labor-intensive and time-consuming nature of manual power imaging analysis. These methods usually include source localization, clustering, averaging, and automatic spike identification, followed by expert assessment. Such pipelines have been shown to exhibit respectable sensitivity and consistency for localizing epileptiform activity under controlled conditions, although their effectiveness is still reliant on high-quality recording and professional supervision [77]. These techniques enable standardized evaluations of source-level epileptiform patterns that may be pertinent for interpreting treatment response in longitudinal or comparative study designs.

Invasive recordings have further expanded source localization strategies. Stereo-electroencephalography (SEEG)–based source localization during seizures, incorporating rotational dipole modeling and current density mapping in both time and frequency domains, has been explored as a means of delineating seizure onset patterns [78]. In selected cohorts, these approaches have been reported to show closer correspondence with surgically defined epileptogenic zones than conventional clinician-defined seizure onset zones, suggesting potential value for contextualizing surgical outcomes rather than for direct clinical decision-making [79, 80]. In parallel, interictal source–sink connectivity metrics derived from patient-specific intracranial EEG models have been proposed to characterize network-level inhibitory and excitatory dynamics. Preliminary studies suggest that such metrics may provide complementary information relevant to surgical outcome assessment when compared with traditional electrophysiological markers, although external validation remains limited [81].

More recently, source-level FC analyses have been applied to identify putative epileptogenic-zone biomarkers in drug-resistant epilepsy and to support machine-learning–based modeling using brief interictal recordings [82]. Time-frequency hybrid networks and other deep learning architectures have been presented as proof-of-concept frameworks for combining spectral and temporal information from intracranial EEG data [83]. Although these methods demonstrate the potential of sophisticated computational models for characterizing epileptogenic zones, their clinical applicability is still limited by small sample sizes, inconsistent recording protocols, and the requirement for open and goal-oriented validation techniques.

## Treatment monitoring

Longer recording times have been linked to a higher diagnostic yield in some clinical contexts, and long-term video-EEG monitoring presents an extended temporal window for catching seizure-related electroclinical events [84]. Scalable analytical techniques for describing seizures, interictal spikes, and spike clusters over extended recordings, as well as signal-processing techniques for identifying rhythmic oscillations and ictal patterns, have been extensively investigated in recent methodological advances [85, 86]. In this regard, QEEG spectrograms have been investigated as supplementary instruments to facilitate the electroclinical distinction between epileptic and non-epileptic events during video-EEG monitoring. QEEG spectrograms are usually produced via continuous time-frequency decomposition of EEG signals and shown as synchronized power-frequency maps aligned with the raw EEG traces and video recordings in modern commercial long-term video-EEG systems [87]. These spectrograms help identify ictal and peri-ictal spectral changes by enabling clinicians to see the temporal evolution of frequency-specific power in real time or during offline review. Although claimed sensitivity and specificity values vary across cohorts and analytical pipelines, time-frequency representations may help electroclinical interpretation when paired with traditional review, according to several studies [88]. Similarly, certain sleep-related discharge patterns have been identified in analyses of prolonged EEG recordings, including overnight data, which may be linked to a subsequent seizure recurrence [89]. A variety of QEEG markers for longitudinal evaluation have been proposed in recent work, including both data-driven machine learning-based features and hypothesis-driven connectivity metrics [90–92]. Instead of replacing of expert judgment, these methods are meant to help physician interpretation by summarizing intricate temporal dynamics throughout lengthy recordings. Currently, methodological heterogeneity, a lack of external validation, and variations in recording techniques limit their therapeutic relevance, highlighting the necessity of careful interpretation in treatment monitoring scenarios [89].

## Neuromodulatory and non-pharmacological therapies

### Neuromodulation-based therapies

Neuromodulation represents a major non-pharmacological strategy in epilepsy treatment, and QEEG has been increasingly applied to characterize treatment-related neurophysiological changes and explore potential biomarkers of therapeutic response. Across different neuromodulatory modalities, QEEG findings generally reflect alterations in cortical synchronization and large-scale network organization, although their clinical interpretability remains variable.

Among available approaches, vagus nerve stimulation (VNS) is the most extensively studied. Across studies, VNS is generally associated with reduced cortical synchronization and shifts in spectral power toward higher frequencies, suggesting desynchronization of epileptic networks [93–96]. These effects are thought to be mediated, at least in part, by neuromodulatory pathways involving the locus coeruleus and dorsal raphe nuclei [97, 98]. However, reported QEEG changes are not entirely consistent, and their relationship to clinical response appears to be context-dependent. For example, responders have been described as exhibiting lower global synchronization and relative increases in higher-frequency activity [94, 99], while intracranial EEG studies indicate region-specific reductions in FC in patients with favorable outcomes [100]. Taken together, these findings suggest that QEEG captures neurophysiological correlates of VNS effects, but lacks specificity and reproducibility as a predictive biomarker.

Non-invasive neuromodulatory techniques, including transcranial direct current stimulation (tDCS) and repetitive transcranial magnetic stimulation (rTMS), have also been evaluated using QEEG. Available evidence suggests that these interventions may modulate interictal epileptiform activity and network-level synchronization. However, current findings are derived primarily from small-scale studies with heterogeneous protocols, limiting the robustness and generalizability of reported effects [101–105].

In contrast to these open-loop approaches, closed-loop neurostimulation represents a more direct integration of quantitative electrophysiological analysis into therapeutic decision-making. In order to identify seizure-related activity and provide response stimulation meant to halt the progression of seizures, these devices continuously evaluate electrocorticographic signals in real time. From a methodological standpoint, this paradigm strongly aligns with the ideas of QEEG since it is based on quantitative signal processing, which includes feature extraction, pattern identification, and algorithm-driven control [106, 107]. State-dependent and on-demand stimulation techniques may enhance seizure control while reducing unnecessary stimulation, according to emerging research [108, 109]. To improve customized treatment responses, machine learning (ML)-based optimization of stimulation parameters has also been investigated [107, 110]. According to clinical research, responsive neurostimulation can reduce seizures in people with drug-resistant epilepsy for an extended period of time [111]. However, these methods may not be as generalizable or comparable to scalp EEG-based QEEG metrics because they mainly rely on intracranial recordings and device-specific algorithms.

Similar network-level effects are shown by deep brain stimulation (DBS), which can be measured using metrics derived from QEEG. The idea that DBS regulates large-scale brain networks is supported by studies that show frequency-specific changes in FC after thalamic stimulation [112, 113]. Nevertheless, results continue to vary and are impacted by stimulation parameters, target selection, and analytical techniques, just like with other neuromodulatory strategies.

Hence, QEEG presents a sensitive framework for identifying alterations in brain activity linked to neuromodulation, especially at the network level. Nevertheless, these measurements exhibit significant variation, poor repeatability, and inadequate validation as predictive biomarkers across modalities. Currently, their main utility is in assisting with mechanistic interpretation rather than directing customized therapy choices.

### Metabolic therapy: ketogenic diet

The ketogenic diet (KD) is a metabolically driven treatment approach with unique neurophysiological effects that go beyond neuromodulation. QEEG investigations mainly record downstream electrophysiological changes rather than mechanism-specific signals, despite the fact that several mechanisms have been hypothesized, such as regulation of inhibitory–excitatory balance and mitochondrial activity [114, 115].

Across studies, KD is generally associated with reductions in epileptiform activity and alterations in background rhythms, including changes in alpha-band power and network connectivity patterns that have been linked to clinical improvement, particularly in pediatric populations [116–123]. Network-based analyses further suggest that KD may induce reorganization of large-scale brain networks across specific frequency bands [124, 125]. However, these findings are highly heterogeneous and influenced by differences in dietary protocols, follow-up duration, and analytical methods, limiting their comparability and interpretability.

### Behavioral and adjunctive therapies

In addition to neuromodulatory and metabolic interventions, QEEG has been applied to assess neurophysiological responses to behavioral and sensory-based therapies, although evidence in this area remains limited. Available studies suggest that non-invasive interventions, such as music-based stimulation, may transiently modulate cortical excitability, as reflected by reductions in epileptiform discharges in selected pediatric populations [126–128].

However, these findings are derived from small and methodologically heterogeneous studies, and their clinical significance remains uncertain. At present, QEEG in this context serves primarily as an exploratory tool rather than a validated measure of treatment efficacy.

### Summary and critical considerations

When taken as a whole, QEEG shows sensitivity to neurophysiological alterations in a wide range of therapy modalities, such as behavioral, metabolic, and neuromodulatory strategies. Nevertheless, a recurring trend emerges in all of these domains: reported results are inconsistent, method-dependent, and frequently derive from small-scale investigations.

One major drawback is that QEEG changes sometimes lack specificity, with comparable changes (such as decreased epileptiform activity or changed connectivity) seen in many clinical settings and treatment regimens. This makes it difficult to interpret these metrics as clear markers of the effectiveness of treatment. Reproducibility and cross-study comparability are further limited by variations in study design, data collection, and analytical techniques.

Hence, although QEEG provides a useful framework for describing treatment-related brain dynamics, its present function is still mainly descriptive and hypothesis-generating. To promote its translation into clinical decision-making, future research should focus on methodological standardization, larger prospective trials, and the discovery of clinically significant and modality-specific biomarkers.

## Non-seizure outcome assessment

The management of epilepsy encompasses cognitive and functional objectives in addition to seizure control. QEEG has been investigated as an objective method for evaluating consequences unrelated to seizures, especially those related to ASMs. While medications like topiramate have been associated with changes in spectral power and background organization, which may be related to cognitive impairment, sedatives like benzodiazepines are often linked to increased beta activity and generalized slowing. Notably, in individuals with epilepsy, memory and executive function have been linked to region-specific EEG characteristics, such as anterior prefrontal theta activity [129].

Additionally, QEEG has been used to describe sleep-related dysfunction, which is a significant factor in quality of life. EEG-based investigations have revealed oscillatory and non-oscillatory indicators suggesting anomalies at the network level [130], as well as variations in rapid eye movement sleep in focal epilepsy [131].

Furthermore, QEEG is useful in epileptic encephalopathies, where cognitive and developmental disabilities are caused by epileptiform activity. Quantitative measurements of background activity, epileptiform discharge burden, and network organization may function as supplementary indicators of therapy response in this setting, where therapeutic objectives go beyond seizure reduction to include normalization of abnormal EEG patterns [132, 133].

Overall, although these findings highlight the potential of QEEG to capture non-seizure outcomes, current evidence remains limited by methodological heterogeneity and a lack of standardized biomarkers linking electrophysiological measures to clinically meaningful functional endpoints.

## Treatment response prediction

QEEG has been investigated for its possible role in predicting therapy response in epilepsy, beyond post-treatment review. Responders and non-responders have been found to differ in baseline or longitudinal EEG parameters, including spectral power distributions, FC patterns, and network-level metrics [134–136]. These results imply that neurophysiological patterns linked to treatment responsiveness may be captured by QEEG. Nevertheless, no reliable predictive pattern has been found, and the stated traits are highly inconsistent.

Exploratory relationships between QEEG characteristics and therapy response have been reported in several epilepsy disorders. For instance, responders to anti-seizure medications in temporal lobe epilepsy have been shown to exhibit changes in spectral power and decreased network connectivity after treatment, and baseline connectivity patterns have been studied as potential predictors [134]. Exploratory models linked to treatment resistance or long-term outcomes in childhood absence epilepsy and infantile epileptic spasms syndrome have been built using network-level metrics obtained from coherence, phase synchronization, and graph-theoretical investigations [137, 138]. Despite these findings, stated predictive performance varies greatly between studies and is frequently based on within-cohort, retrospective analysis, which restricts generalizability.

Additionally, QEEG-based predictive techniques have been used in a variety of therapeutic modalities, such as neuromodulation and epilepsy surgery. In neuromodulatory interventions such as VNS, rTMS, and DBS, preliminary studies suggest that spectral and network-level features may be associated with treatment response, although evidence remains limited and heterogeneous [112, 113, 139]. Similar exploratory models have been proposed in behavioral interventions, such as music-based therapy, and in surgical cohorts, where connectivity-derived metrics have been evaluated in relation to postoperative seizure outcomes [126, 140]. However, these findings are highly context-dependent and often reflect post hoc associations rather than robust prospective predictors.

A consistent limitation across studies is the substantial methodological heterogeneity, including differences in patient populations, outcome definitions, feature selection, and analytical approaches. In addition, many predictive models are developed and evaluated within small, single-center cohorts, raising concerns regarding overfitting and limited external validity. Consequently, reported performance metrics should be interpreted with caution, as they may overestimate real-world predictive utility.

Overall, current evidence suggests that QEEG may provide a useful framework for exploring neurophysiological features associated with treatment response. However, its role in prediction remains preliminary. At present, QEEG-based models should be regarded as exploratory and hypothesis-generating, rather than reliable tools for individualized treatment prediction, pending validation in larger, prospective, and methodologically standardized studies.

## AI-assisted treatment evaluation

With the increasing availability of high-dimensional QEEG features spanning spectral, network, and source domains, AI has emerged as a promising framework for integrating multidimensional electrophysiological information in treatment evaluation. Unlike traditional univariate approaches, AI-based models enable the identification of complex patterns associated with treatment response, including differentiation between responders and non-responders and prediction of post-intervention outcomes.

Current applications of AI-assisted QEEG in epilepsy are heterogeneous, encompassing diverse patient populations (pediatric and adult; focal and generalized epilepsy) and outcome targets, such as anti-seizure medication response [141–143], post-surgical seizure control, and seizure recurrence risk [144–146]. Methodologically, approaches range from conventional ML models using predefined, physiologically interpretable features to deep learning architectures with automated feature extraction, reflecting trade-offs between interpretability, flexibility, and data requirements. Representative studies are summarized in Table 4, though these findings should be interpreted cautiously. Most included studies were single-center cohorts relying on internal validation only, with heterogeneous reference standards primarily based on clinically derived outcomes (e.g., seizure frequency reduction, post-surgical seizure control, or recurrence during follow-up), with several lacking fully specified definitions. Such measures depend on clinical reporting and expert interpretation and are therefore potentially subject to recall bias, incomplete documentation, and inter-observer variability. Notably, none reported benchmarking against human expert performance or inter-rater agreement, and no explicit external validation was described.

**Table 4:** Selected studies on AI–assisted QEEG for epilepsy treatment evaluation

Study	AI Method	Epilepsy Type	Population	QEEG Features	Prediction Target	Reference Standard	Sample Size	Validation Strategy	Reported Performance
Li et al., 2024 [143]	k-Nearest Neighbors (KNN)	Childhood absence epilepsy	Pediatric	Power spectral density; band ratios	Valproate treatment response	Clinical treatment response (definition not fully specified)	25	Internal cross-validation	Accuracy 84.6%; AUC 0.88
Mercier et al., 2024 [144]	Artificial Neural Network (ANN)	Focal epilepsy	Pediatric pre-surgical	Power spectra; entropy measures	Surgical outcome	Post-surgical outcome (not fully specified)	123	Internal validation; performance metrics not fully reported	Model significance reported; quantitative metrics not provided
Sheikh et al., 2024 [145]	Ensemble learning (RF, SVM)	Temporal lobe epilepsy	Adult	Pre-ictal qEEG changes	Post-surgical seizure control	Post-surgical seizure outcome (clinical follow-up; likely Engel classification)	294	Internal validation; external validation not reported	Accuracy > 90%; AUC 0.98
Lemoine et al., 2023 [147]	Traditional ML with feature selection	Mixed epilepsy types	Adult	Conventional qEEG metrics	Seizure recurrence risk	Seizure recurrence within 1 year (clinical follow-up)	Not reported	Not reported	AUC 0.63
Yang et al., 2024 [148]	Convolutional Neural Network (CNN)	Drug-resistant epilepsy	Adult	Nonlinear and time-window features	Post-surgical seizure control	Drug-resistant epilepsy classification (clinical criteria)	> 100	Internal validation	Accuracy > 85%

Abbreviations: RF: Random Forest, SVM: Support Vector Machine, ML: Machine Learning, ANN: Artificial Neural Network, CNN: Convolutional Neural Network, AUC: Area Under Curve. Reported performance metrics are not directly comparable across studies due to heterogeneity in prediction targets, feature sets, sample sizes, and validation strategies. In several studies, only partial performance indicators were reported, reflecting the exploratory nature of current AI-assisted QEEG applications

Despite apparently favorable performance metrics reported in selected studies [145], the current body of evidence remains exploratory and methodologically constrained. Most investigations are based on single-center cohorts with limited sample sizes and rely primarily on internal validation strategies. Key methodological details, including data partitioning procedures, external validation, and robustness to inter-subject variability, are often incompletely reported. These limitations substantially restrict the interpretability and generalizability of reported findings.

From a translational perspective, AI-assisted QEEG approaches should therefore be regarded as hypothesis-generating rather than decision-support tools. Traditional ML models using physiologically interpretable features may offer advantages in transparency and reproducibility, whereas deep learning approaches such as convolutional neural networks (CNNs) remain challenged by data scarcity and limited interpretability in clinical EEG applications [148, 149]. Explainable and automated feature extraction is crucial for clinical translation, according to a number of studies [150–153].

The absence of benchmarking against human specialists is a major drawback. Current research rarely reports inter-rater reliability as a benchmark or evaluates whether AI models predict treatment outcomes on par with physicians (such as neurologists or neurophysiologists). Consequently, it is still challenging to place routinely reported measurements in the context of actual clinical decision-making.

Uncertainty in the reference standards themselves exacerbates this restriction. Because widely used outcome definitions rely on expert interpretation and clinical reporting, they are vulnerable to inter-observer variability, recall bias, and insufficient recording. Changes in interictal epileptiform discharges, ≥50% reduction in seizures, and imaging-based evaluations are some of these definitions.

As a result, rather than solely reflecting objective prediction ability, model success may also reflect the quality of underlying annotations.

The quality of the input data also present issues beyond the definition of the output. Model training is often carried out using preprocessed EEG segments that have had noise and artifacts manually eliminated. Although this enhances signal quality, it may not accurately represent real-world situations and may obscure an important question: can AI systems accurately discriminate between physiological brain activity and non-neural signals? In this regard, model explainability and transparency are crucial for both interpretability and spotting possible dependence on non-physiological characteristics or recording-related confounds during model building [154].

Overall, three interconnected issues continue to limit the clinical utility of AI-assisted QEEG: (1) the absence of benchmarking against expert performance; (2) the use of heterogeneous and perhaps unreliable reference standards; and (3) low tolerance for variations in input data quality. Standardized outcome definitions, inter-rater agreement measurements, thorough external validation, and the creation of explainable models that can guarantee physiologically relevant inference are all necessary to address these problems. AI-assisted QEEG should be viewed as an exploratory tool rather than a therapeutically deployable decision-support system until such advancements are made.

## Limitations

Despite the increasing interest in QEEG applications, there are a number of technical and practical issues with QEEG-based potential biomarkers for assessing epilepsy treatment. First, demographic parameters, especially age, have a significant impact on EEG features [155]. While aging is linked to alterations in interictal epileptiform discharge characteristics and a progressive slowing of background rhythms, developmental EEG patterns in pediatric populations differ significantly from those in adult populations [156, 157]. When assessing treatment-related QEEG changes, these age-related effects restrict cross-study comparability and emphasize the necessity of age-matched normative datasets. Furthermore, the reliability of longitudinal quantitative studies may be further challenged by ambient and recording-related factors that impact EEG stability [158]. Reproducibility and biomarker reliability in particular analytical scenarios may be further impacted by variations in EEG data sources, such as scalp versus intracranial recordings.

Second, there is significant variation in the analytical frameworks used in different studies, especially when it comes to FC and network-based measures. Limited repeatability between cohorts is caused by differences in preprocessing pipelines, reference schemes, connectivity estimators, and graph-theoretical parameters [159, 160]. Furthermore, the interpretation of reported treatment effects may be complicated by residual artifacts like muscle and eye movement contamination, which may have disproportionate effects on high-frequency and connectivity-related metrics [161]. Moreover, finding stable and broadly applicable network biomarkers is made more difficult by the innately nonlinear and nonstationary nature of brain dynamics [162]. For many QEEG measures, formal evaluations of test-retest reliability are still scarce, which further reduces confidence in the stability and generalizability of treatment-related quantitative changes.

Third, while automated preprocessing pipelines and ML-based artifact removal techniques have been developed, and international initiatives by the ILAE and the IFCN have proposed standardized EEG acquisition and reporting guidelines [163, 164], these developments only partially mitigate current sources of variability. The size and demographic variety of currently available normative databases are still restricted, and algorithm performance is still dependent on the type of epilepsy, recording settings, preprocessing decisions, and signal source [164, 165].

Lastly, reliability and generalizability are limited since a large portion of the evidence supporting QEEG-based treatment evaluation comes from small, single-center studies with varied designs and little external validation [166]. Therefore, rather than being final clinical decision-making tools, potential QEEG biomarkers should now be viewed as exploratory and supportive tools.

## Summary and outlook

Current research on the use of QEEG in assessing epilepsy treatment spanning pharmaceutical, surgical, neuromodulatory, and computational domains is summarized in this critical narrative review. From a therapeutic standpoint, the main benefit of QEEG is not that it can replace traditional clinical or electrophysiological evaluation, while it can provide supplementary, long-term data that can help with treatment monitoring and the development of hypotheses in certain clinical settings. The literature as a whole indicates that QEEG-derived metrics can provide supplementary information versus traditional visual EEG interpretation. However, their clinical relevance and maturity differ significantly based on the particular application, degree of standardization, and methodological accuracy. Three major categories can be used to classify current candidate QEEG biomarkers for the evaluation of epilepsy treatments from a translational standpoint.

First, QEEG seems to be the most appropriate use for long-term treatment monitoring out of all those examined. Treatment sensitivity and repeatability are most consistently demonstrated by longitudinal spectral power measurements and quantitative markers of epileptiform activity. When assessed in conjunction with seizure outcomes and the larger clinical context, quantitative evaluation of spectral power changes and epileptiform activity provide objective descriptors of treatment-related brain dynamics and may support clinical follow-up [88]. These techniques typically indicate greater stability and reproducibility among investigations versus more complicated analytical techniques. QEEG performs well in this context as an additional tool instead of as a stand-alone factor in determining treatment choices.

There are encouraging but conflicting data for a second group of applications that includes FC and network-based studies in addition to source-level methods for outcome classification and treatment evaluation. Although there have been reports of correlations between network reconfiguration and treatment response, their routine clinical adoption is currently limited due to significant variation in analysis methods, limited reproducibility, and variability among cohorts. Interpretation may be further complicated by variations in EEG data sources (e.g., scalp vs. intracranial recordings).Third, AI-assisted QEEG methods for customized therapy prediction are still mostly in the exploratory stage. Although a number of studies claim to have strong predictive performance, these results are primarily based on small, single-center datasets with little external validation. These models should now be viewed as instruments for generating hypotheses, and their therapeutic relevance will depend on increased interpretability, transparency, and solid validation across a range of patient populations.

In the future, methodological consolidation will be more important for the effective integration of QEEG into ordinary epilepsy care than the development of increasingly sophisticated analytical approaches. Standardized EEG acquisition and preprocessing procedures, the growth of normative datasets that are age and sex matched, strict validation frameworks, and smooth integration into clinical workflows are among the top priorities. To determine where QEEG can accurately guide therapy evaluation and where its role should stay limited to research settings, further multicenter, longitudinal studies focused on clinically significant and interpretable endpoints will be crucial.

The expenses of software, staff training, and analysis time associated with using QEEG in routine practice must be balanced against the incremental clinical benefit, especially when compared to traditional visual EEG assessment in typical clinical settings.

The clinical efficacy of QEEG in the assessment of epilepsy treatment appears to be task-dependent based on the evidence currently discussed here. While their role in presurgical evaluation and outcome stratification is still restricted to specific research settings, QEEG-derived spectral measures and epileptiform activity indices (EAI) consistently support longitudinal treatment monitoring and adjunctive assessment of anti-seizure medication effects. Although there is currently insufficient external validation and standardization to allow routine clinical decision-making, network-based metrics, source-level studies, and AI-assisted models provide possibilities for prediction. Accordingly, QEEG should presently be viewed as a complementary tool whose clinical applicability varies across treatment contexts, rather than as a universal solution for treatment selection or prognostication.

## Data Availability

No datasets were generated or analyzed during the current study; therefore, data sharing is not applicable.

## Abbreviations

AI: Artificial Intelligence
ANN: Artificial Neural Network
ASMs: Anti-seizure Medications
AUC: Area Under Curve
α: Alpha
β: Beta
CNN: Convolutional Neural Network
DBS: Deep Brain Stimulation
δ: Delta
EAI: Epileptiform Activity Indices
EEG: Electroencephalography
FC: Functional Connectivity
FFT: Fourier Transform
γ: Gamma
HFOs: High-Frequency Oscillations
IFCN: International Federation of Clinical Neurophysiology
ILAE: International League Against Epilepsy
KD: Ketogenic Diet
KNN: k-Nearest Neighbors
MEG: Magnetoencephalography
ML: Machine Learning
MRI: Magnetic Resonance Imaging
NTM: Network Topology Metrics
PSA: Power Spectrum Analysis
QEEG: Quantitative Electroencephalography
RF: Random Forest
rTMS: Repetitive Transcranial Magnetic Stimulation
SC: Signal Complexity
SEEG: Stereo-Electroencephalography
SVM: Support Vector Machine
tDCS: Transcranial Direct Current Stimulation
θ: Theta
VNS: Vagus Nerve Stimulation

## References

[1] Feyissa AM, Tatum WO, Adult EEG. Handb Clin Neurol. 2019;160:103–24. 10.1016/B978-0-444-64032-1.00007-2 31277842

[2] Beniczky S, Aurlien H, Brogger JC, Hirsch LJ. Standardized computer-based organized reporting of EEG: SCORE - second version. Clin Neurophysiol. 2017;128(11):2334–46. 10.1016/j.clinph.2017.07.418 28838815

[3] Weber D. EEG in epilepsy. Continuum (Minneap Minn). 2025;31(1):38–60. 10.1212/cont.0000000000001526 39899095

[4] Caeira MW, Caboclo LO, Paola L. An appraisal to Hans Berger by the time of his 150th birthday: the human EEG and tales of blood flow, heat and brain waves. Arq Neuropsiquiatr. 2023;81(12):1163–8. 10.1055/s-0043-1777114 PMC10756801 38157882

[5] Noachtar S, Remi J, Kaufmann E. Electroencephalography - an update. Klinische Neurophysiologie. 2022;53(4):243–52.

[6] Ball T, Kern M, Mutschler I, Aertsen A, Schulze-Bonhage A. Signal quality of simultaneously recorded invasive and non-invasive EEG. NeuroImage. 2009;46(3):708–16. 10.1016/j.neuroimage.2009.02.028 19264143

[7] Klonowski W, Jernajczyk W, Niedzielska K, Rydz A, Stepien R. Quantitative measure of complexity of EEG signal dynamics. Acta Neurobiol Exp (Wars). 1999;59(4):315–21. 10.55782/ane-1999-1316 10645636

[8] Segovia-Oropeza M, Rauf EHU, Heide EC, Focke NK. Quantitative EEG signatures in patients with and without epilepsy development after a first seizure. Epilepsia Open. 2025;10(2):427–440. 10.1002/epi4.13128 PMC12014921 40040314

[9] van Straaten EC, Stam CJ. Structure out of chaos: functional brain network analysis with EEG, MEG, and functional MRI. Eur Neuropsychopharmacol. 2013;23(1):7–18. 10.1016/j.euroneuro.2012.10.010 23158686

[10] Chen M, Guo K, Lu K, Meng K, Lu J, Pang Y, et al. Localizing the seizure onset zone and predicting the surgery outcomes in patients with drug-resistant epilepsy: a new approach based on the causal network. Comput Methods Programs Biomed. 2025;258:108483. 10.1016/j.cmpb.2024.108483 39536406

[11] Aydin S. Determination of autoregressive model orders for seizure detection. Turkish J Electr Eng Comput Sci. 2010;18(1): 23–30.

[12] Aydin S, Saraoglu HM, Kara S. Log energy entropy-based EEG classification with multilayer neural networks in seizure. Ann Biomed Eng. 2009;37(12):2626–30. 10.1007/s10439-009-9795-x 19757057

[13] Skoric T, Djermanovic M, Spasojevic S, O’Toole JM. Machine learning for short-term seizure forecast using neonatal EEG. Annu Int Conf IEEE Eng Med Biol Soc. 2025;2025:1–5. 10.1109/EMBC58623.2025.11254950 41335682

[14] Aydin S. Comparison of power spectrum predictors in computing coherence functions for intracortical EEG signals. Ann Biomed Eng. 2009;37(1):192–200. 10.1007/s10439-008-9579-8 18941895

[15] Holler Y, Nardone R. Quantitative EEG biomarkers for epilepsy and their relation to chemical biomarkers. Adv Clin Chem. 2021;102:271–336. 10.1016/bs.acc.2020.08.004 34044912

[16] Kuenkel H, Schweitzer F, Sternberg M, Sternberg P. Power spectrum analysis of higher central nervous rhythms in the human EEG. Electroencephalogr Clin Neurophysiol. 1969;27(7):677. 10.1016/0013-4694(69)91277-2 4187327

[17] Ivanov AA. Methods for calculating EEG-based epileptiform activity index. Epilepsy paroxysmal conditions. 2024;16(4):402–8.(In Russ.)

[18] Greenblatt RE, Pflieger ME, Ossadtchi AE. Connectivity measures applied to human brain electrophysiological data. J Neurosci Methods. 2012;207(1):1–16. 10.1016/j.jneumeth.2012.02.025 PMC5549799 22426415

[19] Tjostheim D, Sandvin O. Multivariate autoregressive feature extraction and the recognition of multichannel waveforms. IEEE Trans Pattern Anal Mach Intell. 1979;1:80–6. 10.1109/tpami.1979.4766878 21868833

[20] Holler Y, Helmstaedter C, Lehnertz K. Quantitative pharmaco-electroencephalography in antiepileptic drug research. CNS Drugs. 2018;32(9):839–48. 10.1007/s40263-018-0557-x PMC6153969 30151652

[21] Holler P, Trinka E, Holler Y, MEEGIPS-a modular EEG investigation and processing system for visual and automated detection of high frequency oscillations. Front Neuroinform. 2019;13:20. 10.3389/fninf.2019.00020 PMC6460903 31024284

[22] Lafta R, Alshaheen H, Wang B, Tao X, Li L, Zhang K et al. Fast fourier transform and ensemble model to classify epileptic EEG signals. In: 2022 IEEE international conference on big data (Big Data). 2022. p. 6745–6.

[23] Guerrero CM, Trigueros AM, Franco JI. Time-frequency EEG analysis in epilepsy: what is more suitable? In: Proceedings of the fifth IEEE international symposium on signal processing and information technology. 2005. IEEE;2005. p. 202–7.

[24] Ashokkumar SR, MohanBabu G, Anupallavi S. A novel two-band equilateral wavelet filter bank method for an automated detection of seizure from EEG signals. Int J Imaging Syst Technol. 2020;30(4):978–93.

[25] Diaz GF, Virues T, San Martin M, Ruiz M, Galan L, Paz L, et al. Generalized background qEEG abnormalities in localized symptomatic epilepsy. Electroencephalogr Clin Neurophysiol. 1998;106:501–7. 10.1016/s0013-4694(98)00026-1 9741749

[26] Wang G, Wang J, Xin C, Xiao J, Liang J, Wu X. Inflammatory response in epilepsy is mediated by glial cell gap junction pathway (Review). Mol Med Rep. 2021;24(1):493. 10.3892/mmr.2021.12132 PMC8127031 33955516

[27] Clemens B, Menes A, Piros P, Bessenyei M, Altmann A, Jerney J, et al. Quantitative EEG effects of carbamazepine, oxcarbazepine, valproate, lamotrigine, and possible clinical relevance of the findings. Epilepsy Res. 2006;70(2-3):190–9. 10.1016/j.eplepsyres.2006.05.003 16765028

[28] Cho JR, Koo DL, Joo EY, Yoon SM, Ju E, Lee J, et al. Effect of levetiracetam monotherapy on background EEG activity and cognition in drug-naive epilepsy patients. Clin Neurophysiol. 2012;123(5):883–91. 10.1016/j.clinph.2011.09.012 22000706

[29] Guo J, Wang D, Ren M, Xiong B, Li Z, Wang X, et al. QPEEG analysis of the effects of sodium valproate on adult Chinese patients with generalized tonic-clonic seizures. Metab Brain Dis. 2014;29(3):801–7. 10.1007/s11011-014-9561-0 24810633

[30] Khachidze I, Gugushvili M, Makashvili M, Maloletnev V. The investigation of EEG specificity in epileptic children during Depakine therapy. Int J Neurosci. 2016;126(10):912–21. 10.3109/00207454.2015.1083991 26290045

[31] Karlov VA, Vlasov PN, Kozhokaru AB, Orlova AS. [The efficacy and tolerability of extended release carbamazepine in adult patients with new-onset epilepsy using epileptiform activity index]. Zh Nevrol Psikhiatr Im S S Korsakova. 2021;121(3):31–8. 10.17116/jnevro202112103131 33834715

[32] Mattia D, Spanedda F, Bassetti MA, Romigi A, Placidi F, Marciani MG. Gabapentin as add-on therapy in focal epilepsy: a computerized EEG study. Clin Neurophysiol. 2000;111(2):311–7. 10.1016/s1388-2457(99)00240-0 10680567

[33] Pellegrino G, Mecarelli O, Pulitano P, Tombini M, Ricci L, Lanzone J, et al. Eslicarbazepine acetate modulates EEG activity and connectivity in focal epilepsy. Front Neurol. 2018;9:1054. 10.3389/fneur.2018.01054 PMC6297144 30619030

[34] Ahn SJ, Kim TJ, Cha KS, Jun JS, Byun JI, Shin YW, et al. Effects of perampanel on cognition and quantitative electroencephalography in patients with epilepsy. Epilepsy Behav. 2021;115:107514. 10.1016/j.yebeh.2020.107514 33328106

[35] Karatza P, Cserpan D, Moser K, Lo Biundo SP, Sarnthein J, Ramantani G. Scalp high-frequency oscillation spatial distribution is consistent over consecutive nights, while rates vary with antiseizure medication changes. Epilepsia. 2025;66(4):1250–9. 10.1111/epi.18250 PMC11997931 39740252

[36] Zweiphenning W, von Ellenrieder N, Dubeau F, Martineau L, Minotti L, Hall JA, et al. Correcting for physiological ripples improves epileptic focus identification and outcome prediction. Epilepsia. 2022;63(2):483–96. 10.1111/epi.17145 PMC9300035 34919741

[37] Besheli BF, Sha Z, Ayyoubi AH, Swamy CP, Henry TR, Worrell GA, et al. Pseudo-HFOs elimination in IEEG recordings using a robust residual-based dictionary learning framework. IEEE J Biomed Health Inf. 2024;29(2):857–69. 10.1109/JBHI.2024.3516613 PMC11971004 40030514

[38] Lamberink HJ, Otte WM, Geerts AT, Pavlovic M, Ramos-Lizana J, Marson AG, et al. Individualised prediction model of seizure recurrence and long-term outcomes after withdrawal of antiepileptic drugs in seizure-free patients: a systematic review and individual participant data meta-analysis. Lancet Neurol. 2017;16(7):523–31. 10.1016/S1474-4422(17)30114-X 28483337

[39] Sivaraju A, Tao A, Jadav R, Kirunda KN, Rampal N, Kim JA, et al. Antiseizure medication withdrawal, risk of epilepsy, and longterm EEG trends in acute symptomatic seizures or epileptic EEG patterns. Neurol Clin Pract. 2024;14(6):e200342. 10.1212/CPJ.0000000000200342 PMC11341085 39185097

[40] Wu X, Ma JJ. Sodium valproate: quantitative EEG and serum levels in volunteers and epileptics. Clin Electroencephalogr. 1993;24(2):93–9. 10.1177/155005949302400211 8500255

[41] Sannita WG, Balestra V, DiBon G, Hassan KM, Rosadini G. Ammonia-independent modifications of the background EEG signal and paradoxical enhancement of epileptic abnormalities in EEG after acute administration of valproate to epileptic patients. Neuropharmacology. 1993;32(9):919–28. 10.1016/0028-3908(93)90148-v 8232792

[42] Urzi Brancati V, Pinto Vraca T, Minutoli L, Pallio G. Polymorphisms affecting the response to novel antiepileptic drugs. Int J Mol Sci. 2023;24(3):2535. 10.3390/ijms24032535 PMC9917302 36768858

[43] Salinsky MC, Oken BS, Morehead L. Intraindividual analysis of antiepileptic drug effects on EEG background rhythms. Electroencephalogr Clin Neurophysiol. 1994;90(3):186–93. 10.1016/0013-4694(94)90090-6 7511500

[44] Frost JD Jr., Hrachovy RA, Glaze DG, Rettig GM. Alpha rhythm slowing during initiation of carbamazepine therapy: implications for future cognitive performance. J Clin Neurophysiol. 1995;12(1):57–63. 7896910

[45] Salinsky MC, Oken BS, Storzbach D, Dodrill CB. Assessment of CNS effects of antiepileptic drugs by using quantitative EEG measures. Epilepsia. 2003;44(8):1042–50. 10.1046/j.1528-1157.2003.60602.x 12887435

[46] Maschio M, Zarabla A, Maialetti A, Sperati F, Dinapoli L, Dispenza S, et al. Lacosamide on background eeg activity in brain tumor-related epilepsy patients: A case series study. Brain Behav. 2018;8(11):e01067. 10.1002/brb3.1067 PMC6236258 30334378

[47] Herkes GK, Lagerlund TD, Sharbrough FW, Eadie MJ. Effects of antiepileptic drug treatment on the background frequency of EEGs in epileptic patients. J Clin Neurophysiol. 1993;10(2):210–6. 10.1097/00004691-199304000-00008 8505414

[48] Huang ZC, Shen DL. Studies on quantitative beta activity in EEG background changes produced by intravenous diazepam in epilepsy. Clin Electroencephalogr. 1997;28(3):172–8. 10.1177/155005949702800310 9241472

[49] Karlov VA, Kozhokaru AB, Vlasov PN, Pushkar TN, Orlova AS. [Dynamics of epileptiform activity, efficacy and tolerability of valproic acid in adults and adolescents with newly-diagnosed epilepsy]. Zh Nevrol Psikhiatr Im S S Korsakova. 2020;120(7):35–43. 10.17116/jnevro202012007135 32790974

[50] Schneebaum-Sender N, Goldberg-Stern H, Fattal-Valevski A, Kramer U. Does a normalizing electroencephalogram in benign childhood epilepsy with centrotemporal spikes abort attention deficit hyperactivity disorder? Pediatr Neurol. 2012;47(4):279–83. 10.1016/j.pediatrneurol.2012.06.009 22964442

[51] Vendrame M, Khurana DS, Cruz M, Melvin J, Valencia I, Legido A, et al. Aggravation of seizures and/or EEG features in children treated with oxcarbazepine monotherapy. Epilepsia. 2007;48(11):2116–20. 10.1111/j.1528-1167.2007.01210.x 17645535

[52] Gelisse P, Genton P, Kuate C, Pesenti A, Baldy-Moulinier M, Crespel A. Worsening of seizures by oxcarbazepine in juvenile idiopathic generalized epilepsies. Epilepsia. 2004;45(10):1282–6. 10.1111/j.0013-9580.2004.19704.x 15461683

[53] Pavlidis E, Rubboli G, Nikanorova M, Kolmel MS, Gardella E. Encephalopathy with status epilepticus during sleep (ESES) induced by oxcarbazepine in idiopathic focal epilepsy in childhood. Funct Neurol. 2015;30(2):139–41. 10.11138/FNeur/2015.30.2.139 PMC4610762 26415787

[54] Pietrzak B, Czarnecka E. Pharmaco-EEG-based assessment of the interaction between ethanol and oxcarbazepine. Pharmacol Rep. 2010;62(2):278–86. 10.1016/s1734-1140(10)70267-x 20508284

[55] Kural MA, Duez L, Sejer Hansen V, Larsson PG, Rampp S, Schulz R, et al. Criteria for defining interictal epileptiform discharges in EEG: a clinical validation study. Neurology. 2020;94(20):e2139–47. 10.1212/WNL.0000000000009439 PMC7526669 32321764

[56] Tatum WO, Mani J, Jin K, Halford JJ, Gloss D, Fahoum F, et al. Minimum standards for inpatient long-term video-EEG monitoring: a clinical practice guideline of the international league against epilepsy and international federation of clinical neurophysiology. Clin Neurophysiol. 2022;134:111–28. 10.1016/j.clinph.2021.07.016 34955428

[57] Pinto LF, Oliveira JPS, Midon AM. Status epilepticus: review on diagnosis, monitoring and treatment. Arq Neuropsiquiatr. 2022;80(5 Suppl 1):193–203. 10.1590/0004-282X-ANP-2022-S113 PMC9491413 35976303

[58] Hanin A, Demeret S, Nguyen-Michel VH, Lambrecq V, Navarro V. Continuous EEG monitoring in the follow-up of convulsive status epilepticus patients: a proposal and preliminary validation of an EEG-based seizure build-up score (EaSiBUSSEs). Neurophysiol Clin. 2021;51(2):101–10. 10.1016/j.neucli.2021.01.006 33642131

[59] Sargolzaei S, Cabrerizo M, Goryawala M, Eddin AS, Adjouadi M. Scalp EEG brain functional connectivity networks in pediatric epilepsy. Comput Biol Med. 2015;56:158–66. 10.1016/j.compbiomed.2014.10.018 25464357

[60] Leitgeb EP, Sterk M, Petrijan T, Gradisnik P, Gosak M. The brain as a complex network: assessment of EEG-based functional connectivity patterns in patients with childhood absence epilepsy. Epileptic Disord. 2020;22(5):519–30. 10.1684/epd.2020.1203 33052105

[61] Royer J, Bernhardt BC, Lariviere S, Gleichgerrcht E, Vorderwulbecke BJ, Vulliemoz S, et al. Epilepsy and brain network hubs. Epilepsia. 2022;63(3):537–50. 10.1111/epi.17171 35092011

[62] Stacey W, Kramer M, Gunnarsdottir K, Gonzalez-Martinez J, Zaghloul K, Inati S, et al. Emerging roles of network analysis for epilepsy. Epilepsy Res. 2020;159:106255. 10.1016/j.eplepsyres.2019.106255 PMC6990460 31855828

[63] Jiang S, Li H, Liu L, Yao D, Luo C. Voxel-wise functional connectivity of the default mode network in epilepsies: a systematic review and meta-analysis. Curr Neuropharmacol. 2022;20(1):254–66. 10.2174/1570159X19666210325130624 PMC9199542 33823767

[64] Cotic M, Zalay O, Valiante T, Carlen PL, Bardakjian BL. Frequency interactions in human epileptic brain. Annu Int Conf IEEE Eng Med Biol Soc. 2011;2011:2057–60. 10.1109/IEMBS.2011.6090380 22254741

[65] Ouyang CS, Chiang CT, Yang RC, Wu RC, Wu HC, Lin LC. Quantitative EEG findings and response to treatment with antiepileptic medications in children with epilepsy. Brain Dev. 2018;40(1):26–35. 10.1016/j.braindev.2017.07.004 28757110

[66] Hermans K, Ossenblok P, van Houdt P, Geerts L, Verdaasdonk R, Boon P, et al. Network analysis of EEG related functional MRI changes due to medication withdrawal in focal epilepsy. NeuroImage Clin. 2015;8:560–71. 10.1016/j.nicl.2015.06.002 PMC4484549 26137444

[67] Pegg EJ, Taylor JR, Keller SS, Mohanraj R. Interictal structural and functional connectivity in idiopathic generalized epilepsy: a systematic review of graph theoretical studies. Epilepsy Behav. 2020;106:107013. 10.1016/j.yebeh.2020.107013 32193094

[68] Duan W, Chen X, Wang YJ, Zhao W, Yuan H, Lei X. Reproducibility of power spectrum, functional connectivity and network construction in resting-state EEG. J Neurosci Methods. 2021;348:108985. 10.1016/j.jneumeth.2020.108985 33164816

[69] Wong PK. Source modelling of the rolandic focus. Brain Topogr. 1991;4(2):105–12. 10.1007/BF01132767 1793684

[70] Plummer C, Litewka L, Farish S, Harvey AS, Cook MJ. Clinical utility of current-generation dipole modelling of scalp EEG. Clin Neurophysiol. 2007;118(11):2344–61. 10.1016/j.clinph.2007.08.016 17889598

[71] Plummer C, Wagner M, Fuchs M, Vogrin S, Litewka L, Farish S, et al. Clinical utility of distributed source modelling of interictal scalp EEG in focal epilepsy. Clin Neurophysiol. 2010;121(10):1726–39. 10.1016/j.clinph.2010.04.002 20457537

[72] Mirkovic N, Adjouadi M, Yaylali I, Jayakar P. 3-d source localization of epileptic foci integrating EEG and MRI data. Brain Topogr. 2003;16(2):111–9. 10.1023/b:brat.0000006335.75534.78 14977204

[73] Duffner F, Freudenstein D, Schiffbauer H, Preissl H, Siekmann R, Birbaumer N, et al. Combining MEG and MRI with neuronavigation for treatment of an epileptiform spike focus in the precentral region: a technical case report. Surg Neurol. 2003;59(1): 40–5; discussion 5–6. 10.1016/s0090-3019(02)00972-2 12633956

[74] Vulliemoz S, Thornton R, Rodionov R, Carmichael DW, Guye M, Lhatoo S, et al. The spatio-temporal mapping of epileptic networks: combination of EEG-fMRI and EEG source imaging. NeuroImage. 2009;46(3):834–43. 10.1016/j.neuroimage.2009.01.070 PMC2977852 19408351

[75] Baldini S, Coito A, Korff CM, Garibotto V, Ndenghera M, Spinelli L, et al. Localizing non-epileptiform abnormal brain function in children using high density EEG: Electric Source Imaging of focal slowing. Epilepsy Res. 2020;159:106245. 10.1016/j.eplepsyres.2019.106245 31846783

[76] Birot G, Spinelli L, Vulliemoz S, Megevand P, Brunet D, Seeck M, et al. Head model and electrical source imaging: a study of 38 epileptic patients. NeuroImage Clin. 2014;5:77–83. 10.1016/j.nicl.2014.06.005 PMC4081973 25003030

[77] Spinelli L, Baroumand AG, Vulliemoz S, Momjian S, Strobbe G, van Mierlo P, et al. Semi-automatic interictal electric source localization based on long-term EEG monitoring: a prospective study. Epilepsia. 2023;64(4):951-961. 10.1111/epi.17460 36346269

[78] Satzer D, Esengul YT, Warnke PC, Issa NP, Nordli DR, Jr. Source localization of ictal SEEG to predict postoperative seizure outcome. Clin Neurophysiol. 2022;144:142–50. 10.1016/j.clinph.2022.08.013 36088217

[79] Hao S, Zhao H, Feng Z, Liu W, Zhang C, Ping H, et al. HD-EEG source imaging with simultaneous SEEG recording in drug-resistant epilepsy. Epilepsia. 2025;66(11):4451–64. 10.1111/epi.18552 PMC12661267 40674110

[80] Abdallah C, Thomas J, Aron O, Avigdor T, Jaber K, Dolezalova I, et al. Visual features in stereo-electroencephalography to predict surgical outcome: a multicenter study. Ann Neurol. 2025;98(3):547–60. 10.1002/ana.27278 PMC12392059 40519108

[81] Gunnarsdottir KM, Li A, Smith RJ, Kang JY, Korzeniewska A, Crone NE, et al. Source-sink connectivity: a novel interictal EEG marker for seizure localization. Brain. 2022;145(11):3901–15. 10.1093/brain/awac300 PMC10200292 36412516

[82] Ntolkeras G, Makaram N, Bernabei M, De La Vega AC, Bolton J, Madsen JR, et al. Interictal EEG source connectivity to localize the epileptogenic zone in patients with drug-resistant epilepsy: a machine learning approach. Epilepsia. 2024;65(4):944–60. 10.1111/epi.17898 PMC11018464 38318986

[83] Sui L, Zhao X, Zhao Q, Tanaka T, Cao J. Hybrid convolutional neural network for localization of epileptic focus based on IEEG. Neural Plast. 2021;2021:6644365. 10.1155/2021/6644365 PMC8100408 34007267

[84] Adenan MH, Khalil M, Loh KS, Kelly L, Shukralla A, Klaus S, et al. A retrospective study of the correlation between duration of monitoring in the epilepsy monitoring unit and diagnostic yield. Epilepsy Behav. 2022;136:108919. 10.1016/j.yebeh.2022.108919 36166879

[85] Anjum SMM, Kaufer C, Hopfengartner R, Waltl I, Broer S, Loscher W. Automated quantification of EEG spikes and spike clusters as a new read out in Theiler’s virus mouse model of encephalitis-induced epilepsy. Epilepsy Behav. 2018;88:189–204. 10.1016/j.yebeh.2018.09.016 30292054

[86] O’Neill NS, Javidan M, Koles ZJ. Identification of the temporal components of seizure onset in the scalp EEG. Can J Neurol Sci. 2001;28(3):245–53. 10.1017/s0317167100001402 11513344

[87] Raghavan A, Wilson A, Wend C, Alexander A, Habela C, Nauen D. Open-source system for millisecond-synchronized continuous video-EEG. Epilepsy Res. 2018;145:27–30. 10.1016/j.eplepsyres.2018.05.010 PMC6087497 29807246

[88] Goenka A, Boro A, Yozawitz E. Assessing quantitative EEG spectrograms to identify non-epileptic events. Epileptic Disord. 2017;19(3):299–306. 10.1684/epd.2017.0921 28721936

[89] Cerulli Irelli E, Leodori G, Morano A, Di Bonaventura C. EEG markers of treatment resistance in idiopathic generalized epilepsy: from standard EEG findings to advanced signal analysis. Biomedicines. 2022;10(10):2428. 10.3390/biomedicines10102428 PMC9598660 36289690

[90] Akbarian B, Sainburg LE, Janson A, Johnson G, Doss DJ, Rogers BP, et al. Association between postsurgical functional connectivity and seizure outcome in patients With temporal lobe epilepsy. Neurology. 2024;103(7):e209816. 10.1212/WNL.0000000000209816 PMC11373675 39226517

[91] Corona L, Tamilia E, Perry MS, Madsen JR, Bolton J, Stone SSD, et al. Non-invasive mapping of epileptogenic networks predicts surgical outcome. Brain. 2023;146(5):1916–31. 10.1093/brain/awac477 PMC10151194 36789500

[92] Bernardo D, Kim J, Cornet MC, Numis AL, Scheffler A, Rao VR, et al. Machine learning for forecasting initial seizure onset in neonatal hypoxic-ischemic encephalopathy. Epilepsia. 2025;66(1):89–103. 10.1111/epi.18163 PMC11742638 39495029

[93] Lanzone J, Boscarino M, Tufo T, Di Lorenzo G, Ricci L, Colicchio G, et al. Vagal nerve stimulation cycles alter EEG connectivity in drug-resistant epileptic patients: A study with graph theory metrics. Clin Neurophysiol. 2022;142:59–67. 10.1016/j.clinph.2022.07.503 35970060

[94] Bodin C, Aubert S, Daquin G, Carron R, Scavarda D, McGonigal A, et al. Responders to vagus nerve stimulation (VNS) in refractory epilepsy have reduced interictal cortical synchronicity on scalp EEG. Epilepsy Res. 2015;113:98–103. 10.1016/j.eplepsyres.2015.03.018 25986196

[95] Zhang Q, Luo X, Wang XH, Li JY, Qiu H, Yang DD. Transcutaneous auricular vagus nerve stimulation for epilepsy. Seizure. 2024;119:84–91. 10.1016/j.seizure.2024.05.005 38820674

[96] Pan L, Wang J, Wu W, Wang Y, Zhu Y, Song Y. Transcutaneous auricular vagus nerve stimulation improves working memory in temporal lobe epilepsy: A randomized double-blind study. CNS Neurosci Ther. 2024;30:e14395. 10.1111/cns.14395 PMC10848055 37553557

[97] Bowles S, Hickman J, Peng X, Williamson WR, Huang R, Washington K, et al. Vagus nerve stimulation drives selective circuit modulation through cholinergic reinforcement. Neuron. 2022;110:2867–85.e7. 10.1016/j.neuron.2022.06.017 PMC10212211 35858623

[98] Wang Y, Zhan G, Cai Z, Jiao B, Zhao Y, Li S, et al. Vagus nerve stimulation in brain diseases: Therapeutic applications and biological mechanisms. Neurosci Biobehav Rev. 2021;127:37–53. 10.1016/j.neubiorev.2021.04.018 33894241

[99] Qin X, Yuan Y, Chen Y, Liao J, Lin S, Yang Z, et al. [Application of scalp electroencephalogram in treatment of refractory epilepsy with vagus nerve stimulation]. Sheng Wu Yi Xue Gong Cheng Xue Za Zhi. 2020;37(4):699–707. (in Chinese) 10.7507/1001-5515.201909002 PMC10319535 32840088

[100] Bartolomei F, Bonini F, Vidal E, Trebuchon A, Lagarde S, Lambert I, et al. How does vagal nerve stimulation (VNS) change EEG brain functional connectivity? Epilepsy Res. 2016;126:141–6. 10.1016/j.eplepsyres.2016.06.008 27497814

[101] Trimble MR, Hesdorffer D, Hecimovic H, Osborne N. Personalised music as a treatment for epilepsy. Epilepsy Behav. 2024;156:109829. 10.1016/j.yebeh.2024.109829 38761451

[102] Luo WY, Liu H, Feng Y, Hao JX, Zhang YJ, Peng WF, et al. Efficacy of cathodal transcranial direct current stimulation on electroencephalographic functional networks in patients with focal epilepsy: preliminary findings. Epilepsy Res. 2021;178:106791. 10.1016/j.eplepsyres.2021.106791 34837824

[103] Tsipourakis A, Antonakakis M, Kaiser F, Rampp S, Kovac S, Kellinghaus C, et al. The effect of multi-channel tDCS on the directed connectivity patterns of a case with focal epilepsy using a multi-feature machine learning evaluation. IEEE 24th Int Conf Bioinf Bioeng (BIBE). 2024:1–8.

[104] Krystal AD, West M, Prado R, Greenside H, Zoldi S, Weiner RD. EEG effects of ECT: implications for rTMS. Depress Anxiety. 2000;12:157–65. 10.1002/1520-6394(2000)12:3<157::AID-DA7>3.0.CO;2-R 11126190

[105] Vlachos I, Kugiumtzis D, Tsalikakis DG, Kimiskidis VK. TMS-induced brain connectivity modulation in genetic generalized epilepsy. Clin Neurophysiol. 2022;133:83–93. 10.1016/j.clinph.2021.10.011 34814019

[106] Bernabei JM, Li A, Revell AY, Smith RJ, Gunnarsdottir KM, Ong IZ, et al. Quantitative approaches to guide epilepsy surgery from intracranial EEG. Brain. 2023;146:2248–58. 10.1093/brain/awad007 PMC10232272 36623936

[107] Chandrabhatla AS, Pomeraniec IJ, Horgan TM, Wat EK, Ksendzovsky A. Landscape and future directions of machine learning applications in closed-loop brain stimulation. NPJ Digit Med. 2023;6(1):79. 10.1038/s41746-023-00779-x PMC10140375 37106034

[108] Anderson DN, Charlebois CM, Smith EH, Davis TS, Peters AY, Newman BJ, et al. Closed-loop stimulation in periods with less epileptiform activity drives improved epilepsy outcomes. Brain. 2024;147(2):521–31. 10.1093/brain/awad343 PMC10834245 37796038

[109] Paschen E, Kleis P, Vieira DM, Heining K, Boehler C, Egert U, et al. On-demand low-frequency stimulation for seizure control: efficacy and behavioural implications. Brain. 2024;147(2):505–20. 10.1093/brain/awad299 37675644

[110] Dan R, Zhang H, Bai J. Closed-loop control of epilepsy based on reinforcement learning. Int J Neural Syst. 2026;36(2):2550074. 10.1142/S0129065725500741 41104903

[111] Frauscher B, Bartolomei F, Baud MO, Smith RJ, Worrell G, Lundstrom BN. Stimulation to probe, excite, and inhibit the epileptic brain. Epilepsia. 2023;64(Suppl 3):S49–61. 10.1111/epi.17640 PMC10654261 37194746

[112] Acerbo E, Jegou A, Lagarde S, Pizzo F, Makhalova J, Trebuchon A, et al. Frequency-specific alterations in brain connectivity induced by pulvinar stimulation. Epilepsia. 2025;66(8):2690–702. 10.1111/epi.18405 PMC12371610 40252213

[113] Johnson KA, Dosenbach NUF, Gordon EM, Welle CG, Wilkins KB, Bronte-Stewart HM, et al. Proceedings of the 11th annual deep brain stimulation think tank: pushing the forefront of neuromodulation with functional network mapping, biomarkers for adaptive DBS, bioethical dilemmas, AI-guided neuromodulation, and translational advancements. Front Hum Neurosci. 2024;18:1320806. 10.3389/fnhum.2024.1320806 PMC10915873 38450221

[114] Qiao YN, Li L, Hu SH, Yang YX, Ma ZZ, Huang L, et al. Ketogenic diet-produced beta-hydroxybutyric acid accumulates brain GABA and increases GABA/glutamate ratio to inhibit epilepsy. Cell Discov. 2024;10(1):17. 10.1038/s41421-023-00636-x PMC10861483 38346975

[115] Mishra P, Singh SC, Ramadass B. Drug resistant epilepsy and ketogenic diet: a narrative review of mechanisms of action. World Neurosurg X. 2024;22:100328. 10.1016/j.wnsx.2024.100328 PMC10914588 38444870

[116] Hallbook T, Kohler S, Rosen I, Lundgren J. Effects of ketogenic diet on epileptiform activity in children with therapy resistant epilepsy. Epilepsy Res. 2007;77(2-3):134–40. 10.1016/j.eplepsyres.2007.09.008 17996423

[117] Remahl S, Dahlin MG, Amark PE. Influence of the ketogenic diet on 24-hour electroencephalogram in children with epilepsy. Pediatr Neurol. 2008;38(1):38–43. 10.1016/j.pediatrneurol.2007.09.002 18054691

[118] Freeman JM, Vining EP, Kossoff EH, Pyzik PL, Ye X, Goodman SN. A blinded, crossover study of the efficacy of the ketogenic diet. Epilepsia. 2009;50(2):322–5. 10.1111/j.1528-1167.2008.01740.x 18717710

[119] Kessler SK, Gallagher PR, Shellhaas RA, Clancy RR, Bergqvist AG. Early EEG improvement after ketogenic diet initiation. Epilepsy Res. 2011;94(1-2):94–101. 10.1016/j.eplepsyres.2011.01.012 PMC3062190 21345653

[120] Carrette E, Vonck K, de Herdt V, Dewaele I, Raedt R, Goossens L, et al. A pilot trial with modified Atkins’ diet in adult patients with refractory epilepsy. Clin Neurol Neurosurg. 2008;110(8):797–803. 10.1016/j.clineuro.2008.05.003 18572306

[121] Schoeler NE, Wood S, Aldridge V, Sander JW, Cross JH, Sisodiya SM. Ketogenic dietary therapies for adults with epilepsy: feasibility and classification of response. Epilepsy Behav. 2014;37:77–81. 10.1016/j.yebeh.2014.06.007 25010319

[122] Hallbook T, Ji S, Maudsley S, Martin B. The effects of the ketogenic diet on behavior and cognition. Epilepsy Res. 2012;100(3):304–9. 10.1016/j.eplepsyres.2011.04.017 PMC4112040 21872440

[123] Su TY, Hung PL, Chen C, Lin YJ, Peng SJ. Graph theory-based electroencephalographic connectivity and its association with ketogenic diet effectiveness in epileptic children. Nutrients. 2021;13(7):2186. 10.3390/nu13072186 PMC8308392 34202047

[124] El-Shafie AM, Bahbah WA, Abd El Naby SA, Omar ZA, Basma EM, Hegazy AAA, et al. Impact of two ketogenic diet types in refractory childhood epilepsy. Pediatr Res. 2023;94(6):1978–89. 10.1038/s41390-023-02554-w PMC10007663 36906721

[125] Hsieh TY, Hung PL, Su TY, Peng SJ. Graph theory-based electroencephalographic connectivity via phase-locking value and its association with ketogenic diet responsiveness in patients with focal onset seizures. Nutrients. 2022;14(21):4457. 10.3390/nu14214457 PMC9659238 36364720

[126] Lin LC, Lee MW, Wei RC, Mok HK, Yang RC. Mozart K.448 listening decreased seizure recurrence and epileptiform discharges in children with first unprovoked seizures: a randomized controlled study. BMC Complement Altern Med. 2014;14:17. 10.1186/1472-6882-14-17 PMC3893543 24410973

[127] Lin LC, Ouyang CS, Chiang CT, Wu HC, Yang RC. Early evaluation of the therapeutic effectiveness in children with epilepsy by quantitative EEG: a model of Mozart K.448 listening–a preliminary study. Epilepsy Res. 2014;108(8):1417–26. 10.1016/j.eplepsyres.2014.06.020 25060994

[128] Paprad T, Veeravigrom M, Desudchit T. Effect of Mozart K.448 on interictal epileptiform discharges in children with epilepsy: a randomized controlled pilot study. Epilepsy Behav. 2021;114(Pt A):107177. 10.1016/j.yebeh.2020.107177 32536440

[129] Hamedi N, Garcia-Salinas JS, Berry BM, Worrell GA, Kucewicz MT. Anterior prefrontal EEG theta activities indicate memory and executive functions in patients with epilepsy. Epilepsia. 2025;66(4):1274–87. 10.1111/epi.18246 PMC11997909 39760669

[130] Schiller K, von Ellenrieder N, Avigdor T, El Kosseifi C, Abdallah C, Minato E, et al. Focal epilepsy impacts rapid eye movement sleep microstructure. Sleep. 2023;46(2):zsac250. 10.1093/sleep/zsac250 PMC9905780 36242588

[131] Latreille V, Corbin-Lapointe J, Peter-Derex L, Thomas J, Lina JM, Frauscher B. Oscillatory and nonoscillatory sleep electroencephalographic biomarkers of the epileptic network. Epilepsia. 2024;65(10):3038–51. 10.1111/epi.18088 39180417

[132] Whyte-Fagundes P, Marafiga JR, Zhu B, Baraban SC. Zebrafish models of developmental epileptic encephalopathy accurately reflect clinical electrographic biomarkers. Epilepsia. 2026;67(2):923–33. 10.1111/epi.18681 PMC12927678 41118267

[133] Galer PD, McKee JL, Ruggiero SM, Kaufman MC, Ojemann WKS, McSalley I, et al. Quantitative EEG biomarkers in the genetic epilepsies and associations with neurologic outcomes. Neurology. 2025;105(8):e214148. 10.1212/WNL.0000000000214148 PMC13446166 40986432

[134] Ricci L, Assenza G, Pulitano P, Simonelli V, Vollero L, Lanzone J, et al. Measuring the effects of first antiepileptic medication in temporal lobe epilepsy: predictive value of quantitative-EEG analysis. Clin Neurophysiol. 2021;132(1):25–35. 10.1016/j.clinph.2020.10.020 33248432

[135] Shrey DW, Kim McManus O, Rajaraman R, Ombao H, Hussain SA, Lopour BA. Strength and stability of EEG functional connectivity predict treatment response in infants with epileptic spasms. Clin Neurophysiol. 2018;129(10):2137–48. 10.1016/j.clinph.2018.07.017 PMC6193760 30114662

[136] Tracy JI, Doucet GE. Resting-state functional connectivity in epilepsy: growing relevance for clinical decision making. Curr Opin Neurol. 2015;28(2):158–65. 10.1097/WCO.0000000000000178 25734954

[137] Canafoglia L, Dettori MS, Duran D, Ragona F, Freri E, Casellato S, et al. Early clinical and EEG findings associated with the outcome in childhood absence epilepsy. Epilepsy Behav. 2019;98(Pt A):273–8. 10.1016/j.yebeh.2019.06.040 31419648

[138] Kanai S, Oguri M, Okanishi T, Miyamoto Y, Maeda M, Yazaki K, et al. Predictive modeling based on functional connectivity of interictal scalp EEG for infantile epileptic spasms syndrome. Clin Neurophysiol. 2024;167:37–48. 10.1016/j.clinph.2024.08.016 39265289

[139] Brazdil M, Dolezalova I, Koritakova E, Chladek J, Roman R, Pail M, et al. EEG reactivity predicts individual efficacy of vagal nerve stimulation in intractable epileptics. Front Neurol. 2019;10:392. 10.3389/fneur.2019.00392 PMC6507513 31118916

[140] Bear JJ, Sargent JL, O’Neill BR, Chapman KE, Ghosh D, Kirsch HE, et al. Spike-associated networks predict postsurgical outcomes in children with refractory epilepsy. J Clin Neurophysiol. 2023;40(2):123–129. 10.1097/WNP.0000000000000876 PMC9124720 34817446

[141] Lee GH, Sung SM, Choi KD, Kim J, Cho JW, Kim SH. Predicting antiseizure medication response in newly diagnosed epilepsy using quantitative EEG and machine learning. Seizure-European J Epilepsy. 2025;130:59–67. 10.1016/j.seizure.2025.05.007 40382856

[142] Zahnert F, van Mierlo P, Habermehl L, Garcia L, Malki K, Knake S. Evaluation of quantitative EEG markers for predicting outcome after the initial treatment with levetiracetam monotherapy in newly diagnosed epilepsy. Epilepsy Behav. 2025;164:110284 10.1016/j.yebeh.2025.110284 39892272

[143] Li SP, Lin LC, Yang RC, Ouyang CS, Chiu YH, Wu MH, et al. Predicting the therapeutic response to valproic acid in childhood absence epilepsy through electroencephalogram analysis using machine learning. Epilepsy Behav. 2025;164:110284. 10.1016/j.yebeh.2024.109647 38232558

[144] Mercier M, Pepi C, Carf-Pavia G, De Benedictis A, Espagnet MCR, Pirani G, et al. Author correction: the value of linear and non-linear quantitative EEG analysis in paediatric epilepsy surgery: a machine learning approach. Sci Rep. 2024;14:18765. 10.1038/s41598-024-69706-8 PMC11322331 39138244

[145] Sheikh SR, McKee ZA, Ghosn S, Jeong KS, Kattan M, Burgess RC, et al. Machine learning algorithm for predicting seizure control after temporal lobe resection using peri-ictal electroencephalography. Sci Rep. 2024;14(1):21771. 10.1038/s41598-024-72249-7 PMC11410994 39294238

[146] Dhondiyal SA, Dimri SC. Detection and treatment of epilepsy disease using EEG signals. In: 2024 international conference on cybernation and computation (CYBERCOM). IEEE, 2024: 251–5.

[147] Lemoine E, Toffa D, Pelletier-Mc Duff G, Xu AQ, Jemel M, Tessier JD, et al. Machine-learning for the prediction of one-year seizure recurrence based on routine electroencephalography. Sci Rep. 2023;13(1):12650. 10.1038/s41598-023-39799-8 PMC10403587 37542101

[148] Yang S, Li S, Wang H, Li J, Wang C, Liu Q, et al. Early prediction of drug-resistant epilepsy using clinical and EEG features based on convolutional neural network. Seizure. 2024;114:98–104. 10.1016/j.seizure.2023.12.009 38118285

[149] Moon PS, Chaudhary NI, Bainalwar PA, Moon VK, Shambharkar SS. Emerging frontiers of artificial intelligence in epilepsy care: a comprehensive overview. In: 2024 2nd DMIHER international conference on artificial intelligence in healthcare, education and industry (IDICAIEI). IEEE, 2024:1–5.

[150] Rathod P, Bhalodiya J, Naik S. Epilepsy detection using Bi-LSTM with explainable artificial intelligence. In: 2022 IEEE 19th india council international conference (INDICON). IEEE, 2022:1–6.

[151] Esha OK, Haque N, Ahmed F, Begum N. A Comprehensive review on epilepsy diagnosis and prognosis using machine learning and deep learning approaches. In: 2022 IEEE international women in engineering (WIE) conference on electrical and computer engineering (WIECON-ECE). IEEE, 2022:159 – 64.

[152] Schirrmeister RT, Springenberg JT, Fiederer LDJ, Glasstetter M, Eggensperger K, Tangermann M, et al. Deep learning with convolutional neural networks for EEG decoding and visualization. Hum Brain Mapp. 2017;38(11):5391–420. 10.1002/hbm.23730 PMC5655781 28782865

[153] Upadhyaya DP, Prantzalos K, Thyagaraj S, Shafiabadi N, G Fernandez-BacaVaca, Sivagnanam S, et al. Machine learning interpretability methods to characterize brain network dynamics in epilepsy. medRxiv. 2023. 10.1101/2023.06.25.23291874.

[154] Tveit J, Aurlien H, Plis S, Calhoun VD, Tatum WO, Schomer DL, et al. Automated interpretation of clinical electroencephalograms using artificial intelligence. JAMA Neurol. 2023;80(8):805–12. 10.1001/jamaneurol.2023.1645 PMC10282956 37338864

[155] Wei L, Mooney C. Pediatric and adolescent seizure detection: a machine learning approach exploring the influence of age and sex in electroencephalogram analysis. Biomedinformatics. 2024;4(1):796–810.

[156] Perinelli A, Assecondi S, Tagliabue CF, Mazza V. Power shift and connectivity changes in healthy aging during resting-state EEG. NeuroImage. 2022;256:119247. 10.1016/j.neuroimage.2022.119247 35477019

[157] Aanestad E, Gilhus NE, Brogger J. Interictal epileptiform discharges vary across age groups. Clin Neurophysiol. 2020;131(1):25–33. 10.1016/j.clinph.2019.09.017 31751836

[158] Sobaniec W, Kulak W, Bielewicz B, Sobaniec P. Annual variations of quantitative EEG in patients with chronic epilepsy. Adv Med Sci. 2008;53(2):321–5. 10.2478/v10039-008-0052-6 19095583

[159] Lachin JM. The role of measurement reliability in clinical trials. Clin Trials. 2004;1(6):553–66. 10.1191/1740774504cn057oa 16279296

[160] Welton T, Kent DA, Auer DP, Dineen RA. Reproducibility of graph-theoretic brain network metrics: a systematic review. Brain Connect. 2015;5(4):193–202. 10.1089/brain.2014.0313 PMC4432917 25490902

[161] Alvarado-Rojas C, Huberfeld G. Artificial intelligence applied to electroencephalography in epilepsy. Rev Neurol (Paris). 2025;181(5):403–10. 10.1016/j.neurol.2025.02.007 40158909

[162] Bucolo M, Grazia FD, Fortuna L, Frasca M, Sapuppo F, Shannahoff-Khalsa D. Complementary methods for interpreting brain signals: linear versus nonlinear techniques. In: 2007 29th annual international conference of the IEEE engineering in medicine and biology society. IEEE, 2007:1969–72. 10.1109/IEMBS.2007.4352704 18002370

[163] Kalita B, Deb N, Das D. AnEEG: leveraging deep learning for effective artifact removal in EEG data. Sci Rep. 2024;14(1):24234. 10.1038/s41598-024-75091-z PMC11484783 39414897

[164] Janiukstyte V, Owen TW, Chaudhary UJ, Diehl B, Lemieux L, Duncan JS, et al. Normative brain mapping using scalp EEG and potential clinical application. Sci Rep. 2023;13(1):13442. 10.1038/s41598-023-39700-7 PMC10439201 37596291

[165] Smith G, Stacey WC. The accuracy of quantitative EEG biomarker algorithms depends upon seizure onset dynamics. Epilepsy Res. 2021;176:106702. 10.1016/j.eplepsyres.2021.106702 PMC8504852 34229226

[166] Baker M. 1,500 scientists lift the lid on reproducibility. Nature. 2016;533(7604):452–4. 10.1038/533452a 27225100

